# A Systematic Approach to *Agastache mexicana* Research: Biology, Agronomy, Phytochemistry, and Bioactivity

**DOI:** 10.3390/molecules26123751

**Published:** 2021-06-20

**Authors:** Mariana Palma-Tenango, Rosa E. Sánchez-Fernández, Marcos Soto-Hernández

**Affiliations:** 1 Facultad de Ciencias, Universidad Nacional Autónoma de México, Ciudad Universitaria, Coyoacán, 04510 Ciudad de México, Mexico; marianapt@ciencias.unam.mx; 2 Laboratorio Nacional de Investigación y Servicios Agroalimentarios y Forestales (LANISAF), Edificio Efraím Hernández Xolocotzi Nivel 1 y 2, Universidad Autónoma Chapingo, 56230 Texcoco, Mexico; resf2012@gmail.com; 3 Posgrado en Botánica, Colegio de Postgraduados-Campus Montecillo, km 36.5, Carretera México-Texcoco, 56230 Texcoco, Mexico

**Keywords:** toronjil, Mexican agastache, aromatic plants

## Abstract

Mexico is the center of origin of the species popularly known as toronjil or lemon balm (*Agastache mexicana* Linton & Epling). Two subspecies have been identified and are commonly called purple or red (*Agastache mexicana* Linton & Epling subspecies. mexicana) and white (*Agastache mexicana* subspecies xolocotziana Bye, E.L. Linares & Ramamoorthy). Plants from these subspecies differ in the size and form of inflorescence and leaves. They also possess differences in their chemical compositions, including volatile compounds. Traditional Mexican medicine employs both subspecies. *A. mexicana* exhibits a broad range of pharmacological properties, such as anti-inflammatory, anxiolytic, and antioxidant. A systematic vision of these plant’s properties is discussed in this review, exposing its significant potential as a source of valuable bioactive compounds. Furthermore, this review provides an understanding of the elements that make up the species’ holistic system to benefit from lemon balm sustainably.

## 1. Introduction

Lamiaceae is the eighth most diverse plant family in Mexico and 5.5% of the species worldwide are found in this country. Thus, Mexico may be one of the most important diversification centers [1]. This family contains a wide range of aromatic plants possessing agronomical, pharmacological, and commercial potential. Mexican *Agastache* belongs to this family and its use and commercialization for traditional Mexican medicine make it the most important member of the *Agastache* genus in Mexico [2,3]. The species *Agastache mexicana* divides into two subspecies, based on anatomical characteristics [2] and chemical composition [4]: red lemon balm, *Agastache mexicana* Linton & Epling subspecies mexicana, and white toronjil, *Agastache mexicana* subspecies xolocotziana Bye, E.L. Linares & Ramamoorthy [5].

**Figure 1:**
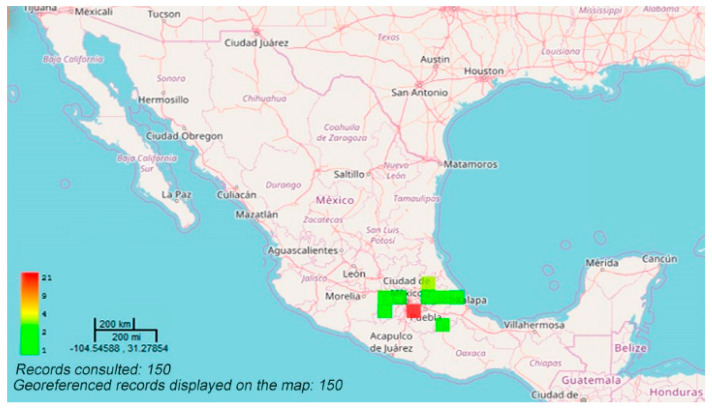
Distribution of *Agastache mexicana* in Mexico [7].

The species is distributed in the states of Guanajuato, Mexico, Michoacán, Puebla, Querétaro, Hidalgo, Veracruz, Chihuahua, Morelos, and Tlaxcala, as well as in Mexico City (Figure 1). The species concentrates in the volcanic axis of Central Mexico [2,6]. 

This article presents a review of the research on *Agastache mexicana* from the perspective of a holistic system to understand its components and interactions. A literature search was sourced from meta-analyses of available *Agastache* data. Data were sourced from bibliographic engines like Web of Science^®^, Scopus^®^, ScienceDirect^®^, and Google Scholar^®^ using “*Agastache mexicana*” as a keyword. 

## 2. A Holistic Approach to *Agastache mexicana* Usage

Systems biology analysis allows the understanding of different biological elements and their interactions with non-biological elements, such as the environment or human impacts (for example, the analysis of various traditional medicine systems like traditional Chinese medicine) [8]. Systems biology, in tandem with reverse pharmacology, may allow discovering new active biological compounds [9]. 

Life science studies relying on systems biology and holistic approaches shy away from reductionist views and incorporate biological effects and their interaction with the environment [8,9]. A biological system contains numerous components interacting in a vast variety of combinations. Once the components and interactions of a system are known, a system’s behavior may be understood [10]. 

**Figure 2:**
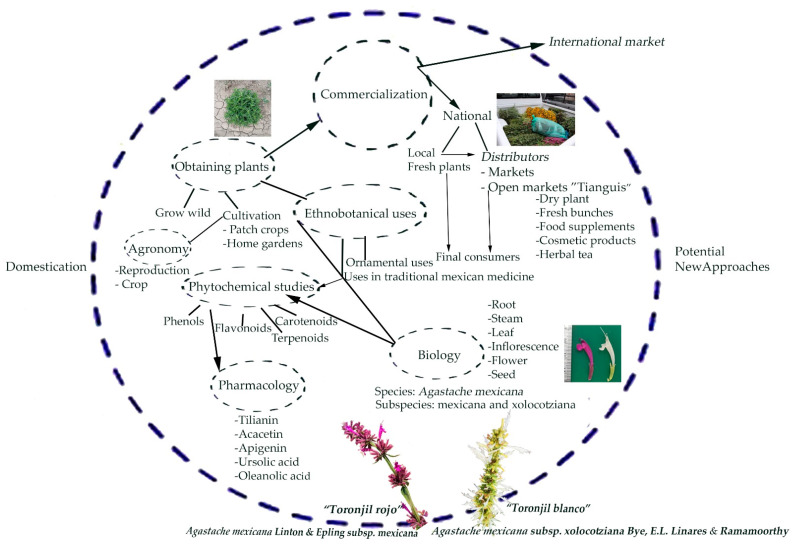
Systems biology approaches of the study of *Agastache mexicana*.

We used systems biology principles for a holistic analysis of different components within the lemon balm plant system and its environment (Figure 2). This system’s insights are derived from a general vision that includes the system components’ relationships and interactions. This approach may provide new collaborative information, fresh insights, and research prospects for the species.

### 2.1. Biology

*A. mexicana* is a native vascular plant of Mexico [11]. It is a perennial herb. Plants of both subspecies have a typical Lamiaceae morphology: opposite, petiolate leaves, a four-angled stem, and numerous trichomes [12]. In the two subspecies, the stem is quadrangular in cross section; in *A. mexicana* ssp. mexicana, the basal and middle part of the stem is purple. The cuticle is smooth, but in the angles, it is observed to be crenulated, with a thickness of 4–8 μm. The three types of epidermal appendages described for the leaf are observed, but uniseriate non-glandular trichomes are abundant in *A. mexicana* ssp. xolocotziana, while they are more scattered and not visible to the naked eye in *A. mexicana* ssp. mexicana [2]. The plant height reaches between 50 and 150 cm [2] and the base chromosome number is 9. The plants have slender and spreading rhizomes [5].

Various plants from the *Agastache* genus are used for bee forage and honey production [13,14]. Toronjil is a honey plant; its flowers produce nectar for bee collection [15].

#### 2.1.1. *A. mexicana* ssp. mexicana

**Figure 3:**
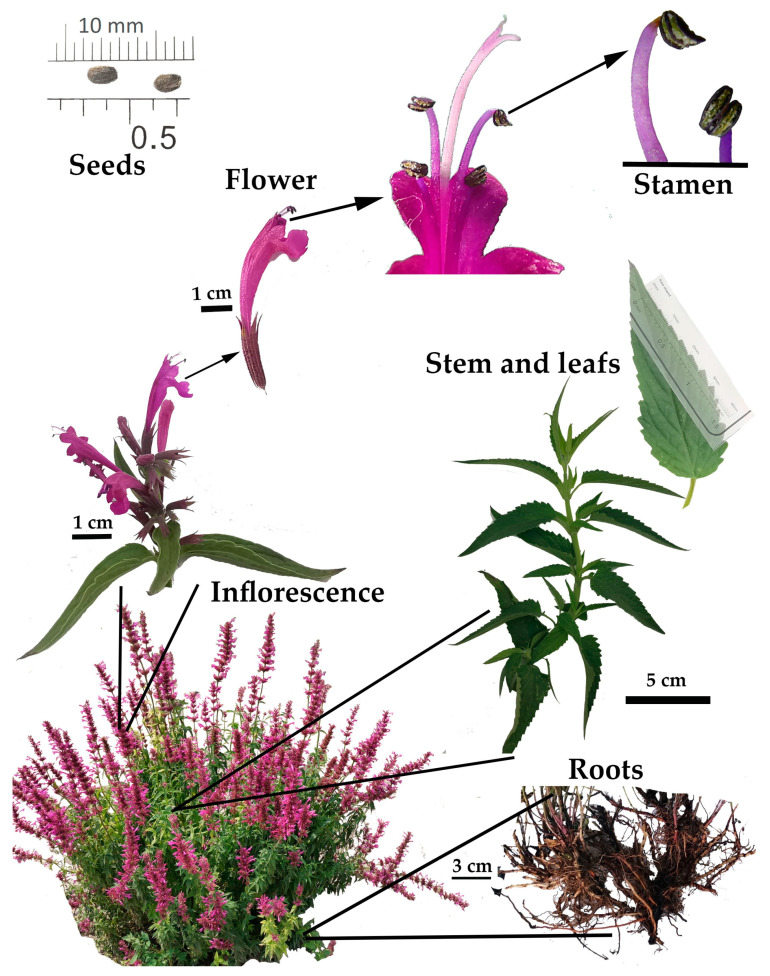
Plant biology of *Agastache mexicana* ssp. mexicana.

The stem of *A. mexicana* ssp. mexicana is erect, branched, and four-angled. The basal and middle part of the stem is purple. The form of the leaves is ovate-lanceolate, measuring 4.4 to 6.3 cm long and 2.1 to 2.5 cm wide [2]. The petiole is 1 cm long [2], and the corolla is purplish red to red [2,5]. The seeds measure approximately 4 to 5 mm (Figure 3).

#### 2.1.2. *A. mexicana* ssp. xolocotziana

**Figure 4:**
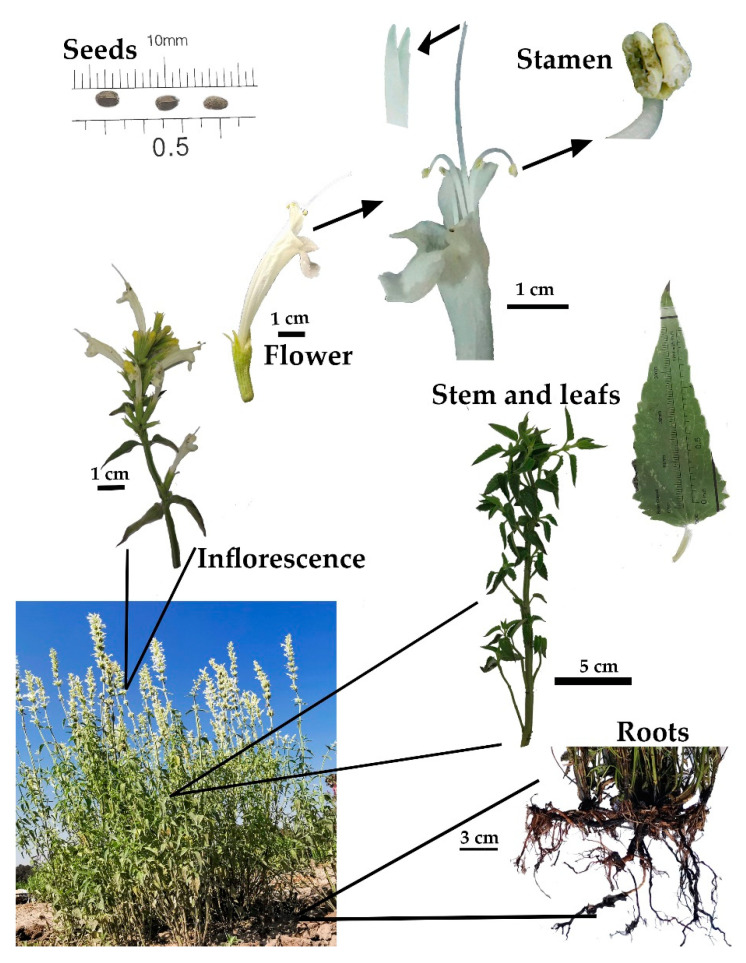
Plant biology of *Agastache mexicana* ssp. xolocotziana.

The stem of *A. mexicana* ssp. xolocotziana is erect, branched, and four-angled. The form of the leaves is ovate-lanceolate, measuring 4.6 to 6.2 cm long and 1.7 to 3.0 cm wide [2]. The petiole is 1 cm long [2]. Inflorescences end in ramifications of interrupted whorls of cymes with numerous flowers. The calyx is 1.0 to 1.3 cm long and the corolla is white and approximately 2.4 cm long. Stamens are didynamous and exserted. Its anther is approximately 1 mm long. The style is 2.8 cm long, and its tip is bifid with the upper arm slightly longer. Ovules are only 0.5 mm tall [5]. The seeds measure approximately 3 to 4 mm (Figure 4).

### 2.2. Ethnobotanical Uses

The genus *Agastache* includes ornamental plants and aromatic plants that contain essential oils [12]. For example, both *A. mexicana* subspecies have therapeutic and ornamental uses [2]. Knowledge about lemon balm healing properties is cited in sources dating back to pre-Hispanic culture, such as in the De la Cruz Badiano Codex [16]. In the Nahuatl tongue, *A. mexicana* is known as tlalahuehuetl [6]. Traditional Mexican medicine labels the plant as “hot”. It is prescribed to cure fright, stomach pain, excessive bile, cough, vomit, chills, and anxiety [2].

The holistic method to study plants with medicinal properties examines the interactions and relationships among the environment’s biological and cultural components. Rural and urban populations use this plant for in-home treatments in the form of herbal teas (infusions and decoctions) [17]. In Mexico, *A. mexicana* is identified for its medicinal properties against anxiety and as a sleep-promoting plant [3,16]. The subspecies have specific uses: *A. mexicana* ssp. mexicana is preferred for wound healing, as an antispasmodic agent, and against stomach pain, while *A. mexicana* ssp. xolocotziana is employed to treat heart disease.

Many modern drugs originated from ethnopharmacology and knowledge of traditional medicine [18]. Results from research on the medicinal effects of *A. mexicana* ssp. mexicana and ssp. xolocotziana support their use in traditional medicine as an anxiolytic, tranquilizer, and sedative, as well as a remedy to alleviate “nervousness” [3,19].

### 2.3. Agronomy

Many medicinal and aromatic plants are industrially sown, but most are still obtained by harvesting wild populations. The need for renewable sources and protection of plant biodiversity creates an opportunity for farmers to grow these crops [20]. In Mexico, over-harvesting of medicinal plants is counteracted by collecting seeds, cuttings, or roots to propagate the plant. Most of these collected samples are planted in small home gardens to be later sown on cultivated fields [21].

*A. mexicana* is a candidate species for structured cultivation as a source for active principles, extracts, essential oils, and pharmaceutical products [22]. Propagation is mainly asexual [23], through vegetative propagation, and depends on its rhizomes’ division, as seed viability is low; *A. mexicana* ssp. xolocotziana seems to have even lower viability. A further complication arises as seed formation is hindered since harvesting occurs during flowering [5]; inflorescences are the main commercialized product. However, red lemon balm exists in wild populations, unlike white lemon balm. A hybridization process between the Mexican subspecies and *Agastache palmeri* possibly originated *A. mexicana* ssp. xolocotziana [23]. 

*A. mexicana* blooms from June to November [22]. Subspecies show phenotypic differences in leaf shape, flower color, and flavor [2]. Farmers from Santiago Mamalhuazuca (State of Mexico) have empirically gathered knowledge that the xolocotziana subspecies is more susceptible to extreme temperature and humidity. The cultivation of both subspecies begins in April, and the stems and inflorescences are harvested in November. The rhizome promotes stem sprouting, which allows a new harvest in the following February [23]. No technological packages based on crop physiology, detailing handling on its phenological stages, leading to higher biomass yields or providing information on bioactive production per cultivation area, exist for *A. mexicana* cultivation.

### 2.4. Commercialization

Ethnobotanical studies have impacted toronjil research. Empirical observations have detected that mexican markets sold a different subspecies from the typical *A. mexicana* subspecies mexicana. Identification of *A. mexicana* subspecies xolocotziana occurred through differential characterization of morphological, chemical, and pharmacological features [2,5]. The commercialization of botanical products promotes the cultural exchange of traditional knowledge and the exploitation of natural resources. Studies illustrate the influence popular markets have on the demand for plants with novel applications. Attention should also focus on the dangers of overcollection of wild species in response to increasing demand and supporting natural habitats’ conservation [21].

White and red lemon balm are commercially sown and traded in various Mexican regions, including Hidalgo, Mexico, Morelos, Puebla, and Veracruz. Inflorescence bundles or dried plants are distributed through different regional sales channels in the State of Mexico, Southeast Puebla, Morelos, and Mexico City [24]. 

### 2.5. Phytochemical and Biological Activity

#### 2.5.1. Phytochemistry

The *Agastache* genus produces various volatile and non-volatile secondary metabolites, mainly phenylpropanoids and terpenoids. *A. mexicana* contains terpenoid compounds like monoterpenes (limonene, pulegone), sesquiterpenes (*β*-caryophyllene), diterpenes (breviflorine), triterpenes (ursolic, corosolic, maslinic acids); phenolic and phenylpropanoid compounds like flavones (acacetin) and flavonoids (tilianin, hesperitin); carboxylic acids (9-hexadecenoic acid, butanoic acid); and soluble sugars (glucose, sucrose) [12]. Subspecies mexicana and xolocotziana share common compounds, but have different chemical profiles [3,4].

The chemical composition of essential oils is influenced by the subspecies, environmental conditions of the crop, harvest time and type of extraction [4,12]. Table 1 shows the chemical compositions of different essential oils obtained from *A. mexicana* subspecies. However, some chemical studies do not specify the studied subspecies, which makes it difficult to establish a defined chemical profile for each subspecies. Plants introduced to other countries have essential oils with different chemical compositions. For example, the essential oil of *A. mexicana* grown in Scotland (subspecies not specified) is characterized by pulegone as the main compound, followed by menthone and limonene [25]; in contrast, *A. mexicana* ssp. mexicana plants introduced in Belarus produced methyl eugenol and estragole as the main compounds [26]. Extraction methods also influence the variability of the physical and chemical characteristics of the essential oils, but different distillation apparatus does not affect the quality of *A. mexicana* essential oil [27].

Chemical study of aqueous and organic extracts from aerial plant parts and whole plants led to the isolation of monoterpenes, diterpenes, triterpenes, flavones, and flavonoids. Table 2 shows the chemical compositions of non-polar and polar extracts from *A. mexicana* subspecies. The compounds in both subspecies are tilianin, acacetin, ursolic acid, salvigenine, 5-hydroxy-7,4′dimethoxyflavone, (2-acetyl)-7-O-glucosyl acacetin, diosmetin 7-O-β-D-(6″-O-malonyl)-glucoside, acacetin 7-O-β-glucoside, acacetin 7-O-β-D-(6″-O-malonyl)-glucoside, acacetin-7-O-β-glucoside-D-(2″-acetyl-6″malonyl), diosmetin, gardenin A, 5,6,7,8,3- pentahydroxy-4-methoxy flavone, 8-hydroxy-salvigenin, α-terpineol, and pulegone (Table 2). The concentration of each compound varies in both subspecies [3], tilianin and acacetin are more abundant in the subspecies xolocotziana [28].

#### 2.5.2. Biological Activity

The biological activity attributed to *A. mexicana* differs between subspecies because each one has a different chemical profile [4]. It also differs with respect to essential oils or extracts, as well as secondary metabolites present in them. Terpenes and flavonoids, such as ursolic acid, oleanolic acid, acacetin, apigenin, and tilianin, are the most active [33]. In some studies, where the biological activity of *A. mexicana* is determined, the studied subspecies is not specified. Table 1 and Table 3 show the biological activities of the essential oils, extracts, and compounds for each subspecies.

##### Antihypertensive, Vaso-Relaxant, Spasmolytic, and Spasmogenic Properties

Pharmacological studies correlate with the ethnomedicinal uses of *A. mexicana*. In 1982, infusions from both subspecies underwent pharmacological studies. Results showed contrasting effects for each subspecies. It was found that *A. mexicana* ssp. xolocotziana contracted the aorta, bladder, intestinal and uterine muscles, and the heart in experiments with frogs [5]. Studies performed in 2009 and 2010 showed antioxidant and vasoactive activities for *A. mexicana* ssp. mexicana extracts (Table 3) [19]. Additionally, the flavonoid tilianin, extracted from the plant, had antihypertensive and vasorelaxant effects on in vitro experiments, as observed on rat aortic rings and in vivo experiments in spontaneously hypertensive rats (SHR) [35]. A later study validated a liquid chromatographic method to detect tilianin in aqueous and organic (methanolic and hydroalcoholic) extracts of *A. mexicana* ssp. mexicana and correlated the biological activity with tilianin content and extraction conditions. The methanolic extracts had higher concentrations of tilianin and were the more vasorelaxant on thoracic aorta rat rings compared to carbachol, while the methanol extracts from dried biomass at 100, 90, and 50 °C were potent vasorelaxants [40]. Tilianin did not have toxic effects in sub-acute and acute oral administration in mice [36]. The vasorelaxant activity of the dichloromethane soluble extract from *A. mexicana* ssp. mexicana and its components (ursolic acid, oleanolic acid, and acacetin) also showed therapeutic effects: ursolic acid and acacetin had antihypertensive activity [38] (Table 3). 

Furthermore, spasmogenic and spasmolytic activities differ between the two subspecies [28]. *A. mexicana* ssp. mexicana extracts were spasmogenic in guinea pig ileum, while *A. mexicana* ssp. xolocotziana extracts had a spasmolytic effect. Additionally, subs. xolocotziana contains a higher amount of acacetin and tilianin. Thus, only *A. mexicana* ssp. xolocotziana should be used to treat gastrointestinal afflictions [28] (Table 3). These results disagree with those reported in 1982 [5], but confirm each toronjil subspecies’ contrasting pharmacological effects. 

Studies of the effect of *A. mexicana* ssp. xolocotziana hexanic, dichloromethanic and methanolic extracts on tracheal rat rings found a relaxant-like activity [41], while the essential oil of *A. mexicana* ssp. mexicana caused relaxation of guinea pig tracheal tissue. The essential oil contains primarily estragole and D-limonene, which act as relaxants and anti-asthmatic compounds [31] (Table 1 and Table 3). These studies support the potential therapeutic use of *A. mexicana* for asthma treatment.

Plant-tissue cultures of *A. mexicana* ssp. mexicana further confirmed the observed in vivo antihypertensive and vasorelaxant effects in SHR [33,35]. Tilianin isolated from methanolic extracts obtained from in vitro plantlets and calli confirmed the conservation of its vasorelaxant effects. The in vitro methanolic extracts contained a higher concentration of tilianin and produced a stronger vasorelaxant effect on aorta rat rings than extracts from wild plants [33] (Table 3).

##### Analgesic and Anti-inflammatory Properties and Effects in the Central Nervous System

The first pharmacological study on the effects of water-soluble *A. mexicana* extract on the central nervous system showed an anxiogenic-like effect in behavioral experiments at the doses tested in male rats [42]. Chemical and pharmacological studies performed in 2014 to identify the effects aqueous extracts from both subspecies have on the central nervous system found similar chemical profiles but different compound abundances. Low doses of the extracts produced an anxiolytic effect, but higher doses sedated mice. Flavonoid derivatives may be responsible for the observed pharmacological effect [3]. Additionally, organic extracts of *A. mexicana* ssp. xolocotziana contain acacetin and ursolic acid and produce anxiolytic, spasmolytic, and antinociceptive effects in in vitro and in vivo experiments in mice [39]. In vivo experiments in different pain models in rodents confirmed the antinociceptive effect of organic *A. mexicana* ssp. xolocotziana extracts and ursolic acid [24,39]. Further behavioral experiments in mice determined the anxiolytic effects of *A. mexicana* ssp. mexicana methanolic extract and tilianin; they confirmed lemon balm contains tilianin, an anxiolytic compound, plus acacetin and ursolic acid [37] (Table 3). Taken together, results from pharmacological studies validate the traditional use of toronjil (lemon balm) to relieve gastrointestinal disorders, stomach pain, asthma, anxiety, insomnia, and hypertension.

##### Antioxidant and Nutraceutical Properties

Traditional Mexican medicine promotes lemon balm as an herbal product. However, herbal products lack strict quality control to guarantee their chemical composition or authenticity for manufacture. Thus, the consumer may not experience the expected therapeutic effect. However, various herbal products containing *A. mexicana* found significant antioxidant activity [43]. Additional reports detailed similar antioxidant activity of *A. mexicana* [22,34] (Table 3).

*A. mexicana* var. “Sangria” (ssp. mexicana) inflorescences are edible and have nutraceutical potential as they contain sugars and secondary metabolites. Compared to other *Agastache* species and the Lamiaceae family members, lemon balm inflorescences have higher polyphenols and flavonoid content and higher antioxidant properties [22]. 

##### Antifungal and Phytotoxic Properties

Aside from its use as a medicinal plant, *A. mexicana* produces bioactive compounds with antifungal activity (Table 1). The essential oil contains monoterpenes and phenylpropanoids, such as estragole and methyl eugenol, which act as antifungal compounds. Notably, the essential oil did not show toxicity against human macrophages and brine shrimp. Research has shown the potential for its use as a non-toxic botanical fungicidal and as an alternative to synthetic fungicides [32]. A recent study tested the effect of adding *A. mexicana* essential oil to wheat grains as a food preservative for flour and dough. The quality of the dough and cookies prepared with the treated flour did not decrease and the growth of fungal pathogens of *Aspergillus*, *Eurotium*, *Eupenicillum* and *Penicillium* species were reduced (see Table 1). Additionally, by day 49, 79.2% of the added amount persisted. These properties indicate the essential oil as a candidate non-toxic food preservative [44].

In addition, the phytotoxic potential of organic extracts obtained with hexane, acetone and ethanol was explored (Table 3). The acetone extract of *A. mexicana* (subspecies not specified) leaves was the most active, with an IC_50_ of 71 µg/mL on the radical growth of *Amaranthus hypochondriacus* L. [45].

## 3. Potential and Perspectives

The holistic approach to studying the *A. mexicana* species focuses on biology, ethnobotany, chemical composition, and biological activity. The species has potential pharmacological uses as a source of bioactive compounds, such as tilianin, acacetin, apigenin, ursolic acid, and oleanolic acid [33] in areas such as drug development, disease modeling, and other biological explorations [47]. 

Agro-industrial applications and the production of essential oils require greater knowledge and understanding of endemic and native species of cultivated aromatic plants, such as *Agastache mexicana*. It is also essential to develop appropriate technologies for industrial applications and products. The information provided in this review supports the cultivation of lemon balm to take advantage of the plant, extracts, and essential oils. Red lemon balm has high essential oil yields, averaging 2.26%, regardless of the type of distillation device [27], while white lemon balm yields about 1.2%. This essential oil has proven antifungal activity against eleven strains isolated from wheat grains during storage [32]. Extracts from the leaves of *A. mexicana* contain reducing compounds like phenols and flavonoids and have been successfully used to provide a reducing medium for the synthesis of nanoparticles as well as their stability [46].

Progress has been made in the botanical and anatomical differentiation of the two identified subspecies. Evidence from biotechnological studies show that *A. mexicana* plant tissue cultures have great potential as a source of tilianin and other bioactive compounds [33], but information is scarce in terms of a technological package of cultivation and standardization of its components. There is phenotypic variability between subspecies and populations concerning wild or cultivated plants [23]. These results suggest that there may be genetic variability and the potential for genetic improvement of *A. mexicana* to increase plant biomass, improve resistance to climatic factors, resistance to pests and diseases. Furthermore, this variability could allow for the development of populations with specific chemotypes. For this reason, a holistic approach to the study of the species could help visualize a broader panorama that allows the sustainable use of lemon balm. 

## Figures and Tables

**Table 1:** Composition and bioactivity of essential oils from aerial parts of *Agastache mexicana*.

Taxa	Chemical Composition (Main Compound)	Biological Activity
*Agastache mexicana*	Pulegone (75.3%), followed by menthone (13.9%) and limonene (3.1%) [25,29] 17 compounds: menthone (42.4%), isomenthone (18.8%) and pulegone (7.3%) [30]. 44 compounds: pulegone (47.77–49.48%), limonene (15.45–15.93%), *cis*-menthone (12.25–12.89%), and *trans*-menthone (2.89–3.17) [27].	
*Agastache mexicana* Linton & Epling ssp. mexicana	Estragole (86.78%), limonene (11.24%) and linalool (1.98%) [4]. Estragole (80.28%), D-limonene (17.56%) and linalyl anthranilate (17.56%) [31]. 19 volatile compounds ^1^: geranyl acetate (37.5%), followed by geranial (17%) and geraniol (16%) [22]. 16 compounds: geranyl acetate (61.4%) followed by geranial (11%) and geraniol (8.3%) [22]. 21 compounds: estragole (15.1%) and methyl eugenol (20.8%) [26]. Cultivated: estragole, geraniol, linalool, menthone and pulegone. Encouraged: geraniol and pulegone [23].	Tracheal relaxation in guinea pig model. EC_50_ of 18.25 µg mL^−1^ with contractions induced by carbachol and 13.30 µg mL^−1^ with contractions induced by histamine [31].
*Agastache mexicana* ssp. xolocotziana Bye, E.L. Linares & Ramamoorthy	Bornyl acetate [5]. Pulegone (80.07%), limonene (9.49%), menthone (7.91%) and isopulegone (2.53%) [4]. Methyl eugenol (36.41%) estragole (27.92%), linalool (10.66%), menthone (10.29%), pulegone (6.46%) and limonene (5.70%) [32]. Estragole, geraniol, linalool, menthone and pulegone [23].	Antifungal activity (MIC): *Aspergillus amylovorus* (0.3 µg mL^−1^), *A. flavus* (0.3 µg mL^−1^), *A. nomius* (30 µg mL^−1^), *A. ostianus* (30 µg mL^−1^), *Eurotium halophilicum* (30 µg mL^−1^), *Eupenicillum hirayamae* NRRL 3587 (30 µg m^L−1^), *E. hyrayamae* NRRL 3588 (0.3 µg mL^−1^), *E. hyrayamae* NRRL 3589 (30 µg mL^−1^), *E. hyrayamae* NRRL 3591 (0.3 µg mL^−1^), *Penicillium cinnamopurpureum* (0.3 µg mL^−1^), *P. viridicatum* var. ii (30 µg mL^−1^) [32].

^1^ Volatile compounds present in the head space before extraction of the essential oil. EC_50_: mean effective concentration. MIC: minimum inhibitory concentration.

**Table 2:** Chemical composition of aqueous and organic extracts of *Agastache mexicana*.

Taxa	Flavonoids	Flavones	Terpenes	Organic Acids	Esters	Alcohols, Aldehydes, and Ketones	Hydrocarbons
*Agastache mexicana*	Tilianin [33], hesperitin, quercetin [19].		Limonene, linalool, menthone, α-terpineol, pulegone, eugenol [34].				
*Agastache mexicana* Linton & Epling spp. mexicana	Tilianin [28,35,36,37], gardenin A, 5-hydroxy-7,4′ dimethoxy flavone [3].	Acacetin [4,28,38], 7-*O*-glucosyl acacetin, (2-acetyl)-7-*O*-glucosyl acacetin [4], diosmetin 7-*O*-β-D-(6″-*O*-malonyl)-glucoside, acacetin 7-*O*-β-glucoside, acacetin 7-*O*-β-D-(6″-*O*-malonyl)-glucoside, acacetin-7-*O*-β-glucoside-D-(2 ″-acetyl-6″ malonyl), diosmetin, 5,6,7,8,3-pentahydroxy-4-methoxy flavone [3], luteolin 7-*O*-β-D-glucoside, luteolin 7-*O*-β-D-(6-*O*-malonyl)-glucoside [3].	Ursolic acid [4,38], oleanolic acid [38], salvigenine, 8-hydroxy-salvigenin [3], estragole, oleanoic acid [4].	Malic acid [3], hexadecanoic acid, 9-hexadecenoic acid [4].	Butanoic acid- hexane-dioctyl, hexanedioc-dioctyl ester, 6-octen-1-ol- 3,7-dimethyl propionate [4].	3-methoxy-cinnamaldehyde, 2,6-dimethoxy-4-(2-propenyl)-phenol [4]	9-Eicosyne [4]
*Agastache mexicana* spp. xolocotziana Bye, E.L. Linares & Ramamoorthy	Tilianin [28], pratol [5], gardenin A, pilosin [3].	Acacetin [3,4,28,39], 5-hydroxy-7,4′ dimethoxy flavone, (2-acetyl)-7-O-glucosyl acacetin [4], acacetin 7-O-β-glucoside, acacetin 7-*O*-β-D-(6″-*O*-malonyl)-glucoside, acacetin-7-*O*-β-glucoside-D-(2 ″-acetyl-6″-malonyl), diosmetin 7-*O*-β-D-(6 ″ -O-malonyl)-glucoside, diosmetin, 5,6,7,8,3-pentahydroxy-4-methoxy flavone; diosmetin 7-β-O-glucoside, 8-hydroxy-flavone [4], chrysene [5].	Salvigenine, corosolic acid, maslinic acid [4], ursolic acid [4,39], β-amirin, 8-hydroxy-salvigenin [3], breviflorine [5], nerol, pulegone, camphor, *p*-menth-6-ene-2,8-diol, α-terpineol, isopiperitenone, geraniol, α-terpineol-methyl ether, *p*-menthane-1,8-diol, neryl acetate, thymol acetate, piperitone, *p*-menth-2-ene-1,8-diol, isoeugenol, diosphenol, β-terpinyl acetate, ocimenol, 2,8-dihydroxy-*p*-menth-3-en-5-one, *p*-menth-1-en-7,8-diol, linalool 3,7-oxide, oleic acid [4].	Butanoic acid [4].	Hexadecanoic acid methyl ether, ethyl palmitate [4].	2-hydroxy-6-methoxyacetophenone, 2-pentadecanone [4].	9-octadecyne, 3,3,6-trimethyl 1,5-heptadiene [4].

**Table 3:** Biological activity of extracts and compounds isolated from *Agastache mexicana*.

Taxa	Antioxidant	Antimicrobial	Phytotoxic	Central Nervous System	Antihypertensive and Vasorelaxant	Spasmolytic and Antinociceptive
*Agastache mexicana*	Reduction percentage: **Methanol extract:** DPPH ~93%, ABTS ~99%, and TBARS ~94%. **Eugenol:** DPPH ~94%, ABTS ~98%, and TBARS ~98% [34]. **Aqueous extract:** DPPH (IC_50_ 502.3 µg mL^−1^) and TEAC (926.9 µmol Trolox g extract^−1^) [19]. DPPH assay of herbal products containing *A. mexicana*: **Hydroalcoholic extracts** reduction percentage: A, 80.3%; B, 81.4%; C, 80.9%; D, 83.1% [43].	**Aqueous extract** for the synthesis of silver nanoparticles with activity against *Escherichia coli* [46].	Phytotoxic activity at 1000 µg mL^−1^ (% of growth inhibition): **hexane extract** (60.5%) **acetone extract** (85.7%) and **ethanolic extract** (35.5%) on *Amaranthus hypochondriacus* L. **Acetone extract** (48.7%) on *Echinochloa crus-galli* (L.) P Beauv. [45].	**Aqueous extract:** Anxiogenic-like effect in male Wistar rats at doses of 3–12 mg kg^−1^ in elevated plus-maze, forced swimming, and open field tests [42].	Vasorelaxant effect on rat aortic rings: **methanolic extract** of wild plants (Emax = 31.96%, EC_50_ = 113.72 µg mL^−1^), in vitro plantlets (Emax = 37.0%, EC_50_ = 82.64 µg mL^−1^) and callus (Emax = 59.64%, EC_50_ = 105.43 µg mL^−1^) [33]. **Aqueous extract:** EC_50_ 233.7 μg mL^−1^ and Emax 24.9% [19].	
*Agastachemexicana* Linton & Epling ssp. mexicana	DPPH assay of **hydroalcoholic extract:** IC_50_ 1.4 mg mL^−1^ [22].			Anxiolytic effect in mice: **Methanol extract** and **Tilianin** at dosage of 30 mg kg^−1^ (ip.) or 300 mg kg^−1^ (po.) [37]. **Aqueous extract:** activity at low doses (0.1–10.0 mg kg^−1^). Reduced motor coordination and sedative-like actions at high doses (100–200 mg kg^−1^). Toxicity: LD_50_ > 5000 mg kg^−1^ [3].	Vasorelaxant effect in rat aortic rings: **Dichloromethane extract** Emax 76.27%, IC_50_ 189.06 µg mL^−1^ [35]. **Methanolic extract:** Emax 82.3% and EC_50_ 291.25 µg mL^−1^ [40]. **Acacetin:** Emax 63.4% and EC_50_ 210.84 µM. **Ursolic acid:** Emax 86% and EC_50_ 39.56 µM and in vivo antihypertensive action on SHR [38]. **Tilianin** induced NO overproduction in rat aorta: 1.49–0.86 µM of nitrites g^−1^ of tissue and vasorelaxant effect at 0.002–933 µM, Emax 84.7% and EC_50_ 104.4 µg mL^−1^. Antihypertensive action on SHR at 50 mg kg^−1^ [35,40]. LD_50_ of 6624 mg kg^−1^ in mice and antihypertensive effect (ED_50_ 53.51 mg kg^−1^) in SHR [36].	**Methanolic extract:** spasmogenic effect on guinea pig ileum. Maximal contractile response with 316 µg mL^−1^ (60%) [28].
*Agastachemexicana* ssp. xolocotziana Bye, E.L. Linares & Ramamoorthy				Anxiolytic effect in mice: **Acacetin** at dosage of 100–300 mg kg^−1^ in mice [39]. **Aqueous extract** activity at low doses (0.1–10.0 mg kg^−1^). Reduced motor coordination and sedative-like actions at high doses (100–200 mg kg^−1^). Toxicity: LD_50_ of 3807 mg kg^−1^ [3].	Relaxant effect on rat tracheal rings. **Hexane extract:** Emax 100.16% and EC_50_ 219 µg mL^−1^. **Dichloromethane extract:** Emax 97.78% and EC_50_ 320.8 µg mL^−1^. **Methanol extract:** Emax 75.54% and EC_50_ 644.44 µg mL^−1^ [41].	Spasmolytic effect on guinea pig ileum: **Methanolic extract** maximal relaxant effects: 100 µg mL^−1^ (72.6%)–316.2 µg mL^−1^ (68.6%) [28]. **Acacetin** IC_50_ of 1.1 μM and antinociceptive activity in mice (ED_50_ 2 mg kg^−1^). **Ursolic acid:** spasmolytic response and antinociceptive effect: ED_50_ 3 mg kg^−1^ [39] and 2 mg kg^−1^ in mice [24]; ED_50_ 44 mg kg^−1^ in rats [24]. Writhing test in mice: maximum latency at 300 mg kg^−1^ and antinociceptive response of extracts: **hexane** 73% (ED_50_: 56.68 mg kg^−1^), **ethyl acetate** 90% (ED_50_: 31.81 mg kg^−1^), and **methanol** 48% (ED_50_: 253.25 mg kg^−1^). Anti-inflammatory activity on the rat paw and formalin tests. Plantar test: antinociceptive responses of **hexane extract** from 30 to 300 mg kg^−1^ [39].

* DPPH: 1,1-Diphenyl-2-picrylhydrazyl; ABTS: 2,2′-azino-bis (3-ethylbenzothiazoline-6-sulfonic acid), TBARS: thiobarbituric acid reactive substance, TEAC: Trolox equivalent antioxidant capacity, NO: nitric oxide, LD_50_: median lethal dose, IC_50_: mean inhibitory concentration, EC_50_: mean effective concentration, Emax: maximum relaxant effect, ED_50_: median effective dose, SHR: spontaneously hypertensive rats, ip: intraperitoneal injection, po: oral administration.

## Data Availability

Publicly available datasets were analyzed in this study (Figure 1). This data can be found here: https://datosabiertos.unam.mx/biodiversidad/ (accessed on 20 April 2021).
